# Swedish emergency department triage and interventions for improved patient flows: a national update

**DOI:** 10.1186/1757-7241-19-72

**Published:** 2011-12-08

**Authors:** Nasim Farrokhnia, Katarina E Göransson

**Affiliations:** 1 Department of Medical Sciences, Uppsala University, ingång 40, 5 trp, 751 85 Academic Hospital, Uppsala, Sweden; 2 Swedish Council on Health Technology Assessment, Box 3657, S-103 59, Stockholm, Sweden; 3 Department of Emergency Medicine, Karolinska University Hospital Solna, SE-171 76, Stockholm, Sweden; 4 Department of Medicine, Karolinska Institutet, Nobels Väg 13, 171 65 Solna, Sweden

**Keywords:** Emergency department, survey, triage, flow processes

## Abstract

**Background:**

In Scandinavia, emergency department triage and patient flow processes, are under development. In Sweden, the triage development has resulted in two new triage scales, the Adaptive Process Triage and the Medical Emergency Triage and Treatment System. Both these scales have logistic components, aiming to improve patient flows. The aim of this study was to report the development and current status of emergency department triage and patient flow processes in Sweden.

**Methods:**

In 2009 and 2010 the Swedish Council on Health Technology Assessment sent out a questionnaire to the ED managers in all (74) Swedish hospital emergency departments. The questionnaire comprised questions about triage and interventions to improve patient flows.

**Results:**

Nearly all (97%) EDs in Sweden employed a triage scale in 2010, which was an increase from 2009 (73%). Further, the Medical Emergency Triage and Treatment System was the triage scale most commonly implemented across the country. The implementation of flow-related interventions was not as common, but more than half (59%) of the EDs have implemented or plan to implement nurse requested X-ray.

**Conclusions:**

There has been an increase in the use of triage scales in Swedish EDs during the last few years, with acceleration for the past two years. Most EDs have come to use the Medical Emergency Triage and Treatment System, which also indicates regional co-operation. The implementation of different interventions for improved patient flows in EDs most likely is explained by the problem of crowding. Generally, more studies are needed to investigate the economical aspects of these interventions.

## Introduction

When patients can not been seen by a doctor immediately upon arrival to the emergency department (ED), some sort of order for treatment is needed. ED triage, developed since the mid 1900's [1], is nowadays a universal approach for handling such queues [2-4]. Triage is often carried out by registered nurses (RNs) using a triage scale to guide their decision in allocating an acuity level. The development of ED triage varies across the world; Australia being one of the first countries to introduce a five level triage scale, the National Triage Scale (NTS), later renamed the Australasian Triage Scale (ATS) [5]. Anglo-Saxon countries have dominated the development of triage scales, and internationally commonly used scales are the Canadian Emergency Department Triage and Acuity Scale (CTAS), the Manchester Triage Scale (MTS) from the UK and the Emergency Severity Index (ESI) from the US [6-8]. To date, many European countries, e.g. Portugal, the Netherlands and Switzerland, have implemented one of the above mentioned triage scales [9-12].

In Scandinavia, the development of ED triage is rather young. In Denmark, Norway and Finland there are, to our knowledge, only a few studies reporting on ED triage [13,14]. In Sweden, ED triage has attracted attention since the late 1990's, and approximately half of the EDs in a national survey from 2000 reported having RN-led triage [15]. However, more than half of the hospitals stated that they planned to change their triage routine, introducing written guidelines and instructions in order to increase the competency. In 2002, another study, including 87% of all hospital EDs at that time, could not identify the changes planned for in the late 1990's [16]. None of the EDs had implemented any of the internationally developed triage scales, and 46% EDs did not employ a triage scale at all. Several studies have been carried out focusing on triage scales, triage decision making, the triage nurse and the patient perspective of triage e.g. [17-24]. Also, a national network for triage nurses was established 2004, several university courses have been given on the topic, and two new triage scales have been developed, the Adaptive Process Triage (ADAPT) [25] and the Medical and Emergency Triage and Treatment System (METTS) [24].

Both ADAPT and METTS are process-oriented triage scales, and the development of these scales have preceded the development of patient flow processes in Swedish EDs. Currently, many EDs across the country are working towards the British inspired "4-hour goal" [26] but, to the best of our knowledge, no studies have been published to date. Given the broad interest for, and development of, ED triage and patient flow processes in Sweden during the last 10 years, this national survey aims to report the development and current status of ED triage and patient flow processes in Sweden.

## Methods

As part of a governmental assignment carried out by the Swedish Council on Health Technology Assessment, a questionnaire was sent out to the ED managers in all (74) hospital EDs in Sweden the summer of 2009 and autumn of 2010. The 2009 version of the questionnaire contained four questions with multiple choice answers while one question was added to the questionnaire in the 2010 version, hence comprising five questions. This additional question covered the aspect of interventions for improved patient flows used by or planned to be used by hospital EDs by the end of 2011.

## Results

### Usage of triage systems

**Figure 1:**
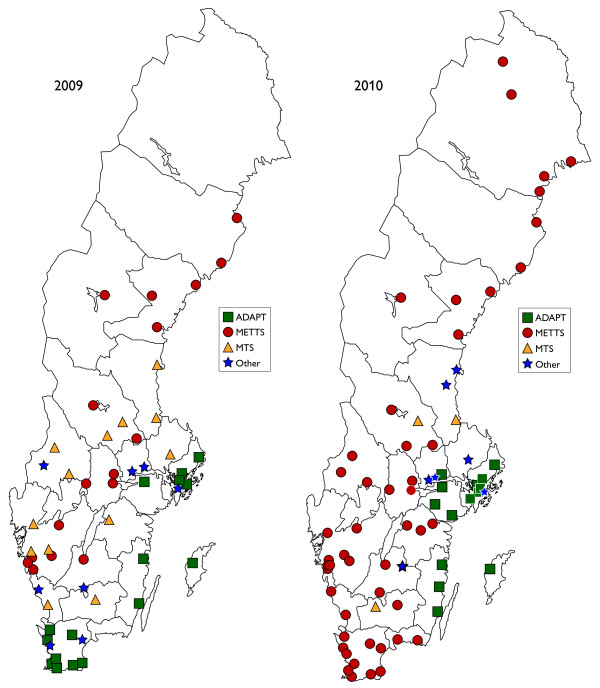
**illustrating the Swedish geographical disposition of the triage scales reported in the study**.

All Swedish EDs participated and returned the questionnaire both in times (2009 and 2010. As seen in Table 1, the proportion of EDs employing a triage scale increased from 73 (2009) to 97 percent (2010). METTS was the triage scale most commonly used at both occasions, and in 2010 nearly two thirds of Swedish EDs employed the scale. As illustrated in Figure 1, METTS was spread across Sweden, with the exception of the south eastern parts of the country. The increase of triage scales occurred all over the country.

**Table 1:** Triage scales used in Swedish EDs (n = 74).

	2009	2010
Triage scales	n (%)	n (%)
ADAPT	15 (20)	14 (19)
Locally developed	9 (12)	7 (9)
METTS	18 (25)	48 (65)
MTS	12 (16)	3 (4)
No scale in use	20 (27)	2 (3)
Total	74 (100)	74 (100)

### Interventions for improved patient flows

Table 2 presents the interventions reported being used to improve patient flows, or planned to be used no later than the end of 2011, by the EDs. The three most common interventions are nurse-requested X-ray, fast track/see and treat and team triage. Triage based streaming, i.e. the sorting of patients based on their triage level instead of their medical speciality, was reported or planned to be used by 16 (22%) EDs.

**Table 2:** Interventions for improved patient flows reported by the 74 EDs.

	2010
Interventions	n (%)
Nurse requested X-ray	44 (59)
Fast track/see and treat	35 (47)
Team triage	32 (43)
Point of care testing	19 (26)
Triage based streaming	16 (22)
Nurse practitioners	10 (14)
X-ray in the ED	10 (14)
CT scan in the ED	1 (1)
Others/none	11 (15)
Total	178*

*EDs could report more than one intervention which is why the sums add up to more than 100%.

Equal amount of EDs (45% respectively) stated that they had/had not evaluated the interventions implemented. Another two (3%) EDs replied that they did not know if any evaluation had taken place while six (8%) EDs refrained from giving information about evaluation.

## Discussion

The main result in this study is that nearly all (97%) EDs in Sweden have introduced triage scales by 2010. Further, METTS is the triage scale most commonly implemented across the country. The implementation of flow-related interventions is not as common, but more than half (59%) of the EDs have implemented or plan to implement nurse-requested X-ray.

Seven years after the first national survey of ED triage scales in Sweden [16], there has been a notable increase in the use of triage scales, from 54 to 97 percent of Swedish EDs employing such a scale. Also, the amount of scales has decreased from 54 to 10, resulting in more EDs with a common platform for triage. This change has taken place without the involvement from national authorities. In the Netherlands, where a national guideline on triage in the EDs was launched in 2004, nearly 39% of the EDs still do not employ a triage scale. Instead, patients are seen in the order of arrival [27].

Based on the results in this study, METTS is the most commonly implemented scale, and with a wide geographic spread. This development has resulted in a common language for clinicians when discussing patient urgency, and facilitates cooperation both within but also outside the ED and the hospital. However, in the perspective of patients' safety, it is important that METTS, as it is the scale most often used, in the near future report high quality studies regarding validity and reliability as there is currently lack of such studies [28]. The reasons for the METTS dominance in Sweden are not known, but one may be the existing cooperation aimed for continuous improvement. Another reason could be the active promotion by the developers.

As METTS is a process-oriented triage scale, and scientific evidence shows that improved flow processes can shorten patient waiting times and lengths of stay in EDs (perhaps without increasing extra costs) [28], there is potential that the implementation of METTS may also have effect on waiting times. To our knowledge there are no published data answering this question, however since the widespread usage of METTS, it would be possible to conduct addressed studies. Originally, ED triage was not developed to decrease waiting time, but to make the waiting time as safe as possible for patients when there is crowding in the ED [29]. The logistic component in ED triage is relatively new, and of the international triage scales, only ESI is based on a logistic component in order to decrease patient waiting time [7]. The flow-related intervention reported to have the best evidence and the potential for the best effect with regards to waiting time and overall length of stay, fast track [28], was reported to be used or planned to be used by nearly half (47%) of the EDs. This can be interpreted as this intervention may be a suitable way of handling one of the most common problems of a crowded ED of our time. Studies show that both patients treated in fast track and those taken care of outside the intervention experience shorter waiting times [28]. The investigations reported in these studies are limited to reorganizing the work and do not include larger budgets or additional staff. However, studies have not specifically investigated the economic aspects of intervention affecting patient flows on ED, why the short- and long-term economic consequences need to be clarified.

On the other hand, it is important when implementing new methods that they are adjusted to the local conditions. Some flow processes could probably be introduced and used in both large and small hospitals around-the-clock (e.g. simple lab specimen analyses in the ED and nurse-requested x-rays) while introducing special, coherent processes (e.g. fast track) might require a certain patient base to optimally utilize resources. Controlled scientific studies have generally been conducted in moderately large to large hospitals, and the flow processes tested have often been used only during daytime hours. If and when they are introduced in Swedish health care, it would appear to be most important to involve large and moderately large hospitals during the periods of the day when loads and staffing levels are highest. Smaller EDs might need to develop specially adapted flow processes, which should include rigorous assessment so the experiences can be shared with other hospitals.

This study is limited to the reporting of a small set of questions regarding ED triage and interventions for improved patient flows. A broader focus such as the triage scales' impact on patient outcome would have added interesting information.

## Conclusions

There has been an increase in the use of triage scales in Swedish EDs during the last few years, with acceleration for the past two years. Most EDs have come to use the Medical Emergency Triage and Treatment System, which also indicates regional co-operation. The implementation of different interventions for improved patient flows in EDs most likely is explained by the problem of crowding. Generally, more studies are needed to investigate the economical aspects of these interventions.

## Competing interests

The authors declare that they have no competing interests.

## Authors' contributions

NF designed the study, developed the questionnaire, carried out the data collection and data analysis as well as wrote the manuscript. KEG designed the study, developed the questionnaire, and wrote the manuscript. Both authors have read and approved the final version of the manuscript
